# Regulation of autophagy by perilysosomal calcium: a new player in β-cell lipotoxicity

**DOI:** 10.1038/s12276-024-01161-x

**Published:** 2024-02-01

**Authors:** Ha Thu Nguyen, Andreas Wiederkehr, Claes B. Wollheim, Kyu-Sang Park

**Affiliations:** 1 Department of Physiology, Yonsei University Wonju College of Medicine, Wonju, Korea; 2 Mitohormesis Research Center, Yonsei University Wonju College of Medicine, Wonju, Korea; 3 Ecole Polytechnique Fédérale de Lausanne, Lausanne, Switzerland; 4 Department of Cell Physiology and Metabolism, University of Geneva, Geneva, Switzerland; 5 Department of Clinical Sciences, Lund University, Malmö, Sweden

**Keywords:** Type 2 diabetes, Energy metabolism

## Abstract

Autophagy is an essential quality control mechanism for maintaining organellar functions in eukaryotic cells. Defective autophagy in pancreatic beta cells has been shown to be involved in the progression of diabetes through impaired insulin secretion under glucolipotoxic stress. The underlying mechanism reveals the pathologic role of the hyperactivation of mechanistic target of rapamycin (mTOR), which inhibits lysosomal biogenesis and autophagic processes. Moreover, accumulating evidence suggests that oxidative stress induces Ca^2+^ depletion in the endoplasmic reticulum (ER) and cytosolic Ca^2+^ overload, which may contribute to mTOR activation in perilysosomal microdomains, leading to autophagic defects and β-cell failure due to lipotoxicity. This review delineates the antagonistic regulation of autophagic flux by mTOR and AMP-dependent protein kinase (AMPK) at the lysosomal membrane, and both of these molecules could be activated by perilysosomal calcium signaling. However, aberrant and persistent Ca^2+^ elevation upon lipotoxic stress increases mTOR activity and suppresses autophagy. Therefore, normalization of autophagy is an attractive therapeutic strategy for patients with β-cell failure and diabetes.

## Autophagy defects: the hidden culprit in diabetes progression

Excessive intake of fatty acids can result in a condition known as lipotoxicity (harmful effect of fats on cells), which can harm pancreatic beta cells (cells that produce insulin) and contribute to type 2 diabetes. Researchers have found that this harm is due to a disturbance in the cells' autophagy process (a system used by cells to eliminate unnecessary or dysfunctional parts). Specifically, the research revealed that the autophagy process is hindered by the activation of a protein complex called mTORC1 and the suppression of a protein named AMPK. These proteins control the autophagy process and their disturbance leads to the buildup of harmful substances in the cell, causing cell death. The researchers propose that future treatment strategies could focus on restoring the balance of these proteins to safeguard pancreatic beta cells from lipotoxicity. This summary was initially drafted using artificial intelligence, then revised and fact-checked by the author.

## Introduction

Diabetes mellitus is a metabolic disease characterized by chronic hyperglycemia due to a deficiency in insulin secretion or an increase in insulin resistance. The main subtype is type 2 diabetes, which is characterized by insulin resistance and impaired insulin secretion. Insulin resistance is triggered by overnutrition and physical inactivity, leading to pancreatic β-cell neogenesis and hypersecretion of insulin to compensate for the elevated insulin demand. However, prolonged exposure to high glucose and saturated fatty acids eventually induces a cytotoxic effect on β-cells, causing defective insulin secretion, a major determinant in disease progression^1,2^.

Pancreatic β-cells play a major role as sensors and rectifiers of glucose homeostasis. Insulin, the main hormone that lowers blood glucose, is secreted from β-cells upon nutrient ingestion. To precisely decode signals reflecting the extracellular metabolic environment, β-cells have a metabolic sensing system. Nutrients are metabolized in the cytosol, and their products funnel into mitochondria to generate ATP and metabolites, which induce insulin exocytosis. Furthermore, upon glucose stimulation, a β-cell produces up to a million molecules of a single-chain precursor, proinsulin, per minute, which is a more than 20-fold increase in protein under the same transcriptional level^3^. Proinsulin enters the endoplasmic reticulum (ER) lumen to undergo protein folding. In the ER lumen, proinsulin molecules acquire three disulfide bonds through prooxidant enzymes such as ER oxidoreductase 1α (ERO1α) and protein disulfide isomerase (PDI), which contribute to reactive oxygen species (ROS) generation under stressful conditions^4^.

Maintenance of functional mitochondria and the ER in β-cells could be threatened by the stress burden related to excess nutrients^5^. This stress causes compensatory increases in insulin synthesis and β-cell proliferation, but prolonged hyperinsulinemia can deteriorate the efficiency of insulin receptor signaling. Insulin resistance can cause β-cell failure due to long-term increased insulin demand^1,2^. Additionally, ROS production induced by lipotoxic conditions can contribute to mitochondrial dysfunction and ER stress, as β-cells have a weak antioxidative capacity to counteract redox insults^6^. Therefore, cellular stresses induced by lipotoxicity impose a double burden on β-cells: an accelerated insulin synthesizing load and cellular oxidative stress, which lead to mitochondrial dysfunction and ER stress.

In addition to stresses on mitochondria and the ER, lysosomal stress can also play a role in β-cell lipotoxicity. Defective autophagic degradation due to lysosomal stress can impair cell survival under lipotoxic stress. The intralysosomal Ca^2+^ concentration is known to be in the range of hundreds of micromolar, corresponding to that of the ER, even though lysosomes do not have any active Ca^2+^ ATPase. This large Ca^2+^ gradient across the lysosomal membrane is known to be dependent on the proton gradient developed by the V-ATPase H^+^ pump^7^. However, Xu et al. suggested that there may exist a direct Ca^2+^ transfer from the ER to lysosomes via passive Ca^2+^ transporters or channels^8^. Disturbance in lysosomal Ca^2+^ homeostasis impairs autophagic degradation and deteriorates cell survival under lipotoxic stress. We have previously reported oxidative stress-mediated ER Ca^2+^ depletion and cytotoxicity by saturated fatty acids^9^, consistent with the results published by others^10^. In this review, we will focus on autophagic defects related to lysosomal Ca^2+^ dysregulation and pathologic signaling in β-cell lipotoxicity, which could be effective therapeutic targets for type 2 diabetes and other metabolic diseases.

## ER calcium depletion in beta-cell lipotoxicity

A large body of evidence describes the involvement of ER stress and mitochondrial dysfunction in β-cell lipotoxicity related to oxidative stress. Elevated levels of glucose and saturated fatty acids elicit pathologic oxidative stress through different mechanisms, such as activation of the protein kinase C (PKC)-NADPH oxidase (NOX) axis in the cytosol or increased superoxide generation from mitochondria. ERO1α and PDI also participate in ROS generation from the ER, which amplifies oxidative stress via a feed-forward mechanism. This redox disequilibrium induces aberrant ER Ca^2+^ release by activating inositol trisphosphate (IP_3_) receptors and ryanodine receptors^11–13^. Oxidative stress also decreases sarco(endo)plasmic reticulum Ca^2+^ ATPase (SERCA) activity^14^, resulting in ER Ca^2+^ depletion and associated ER stress.

**Fig. 1:**
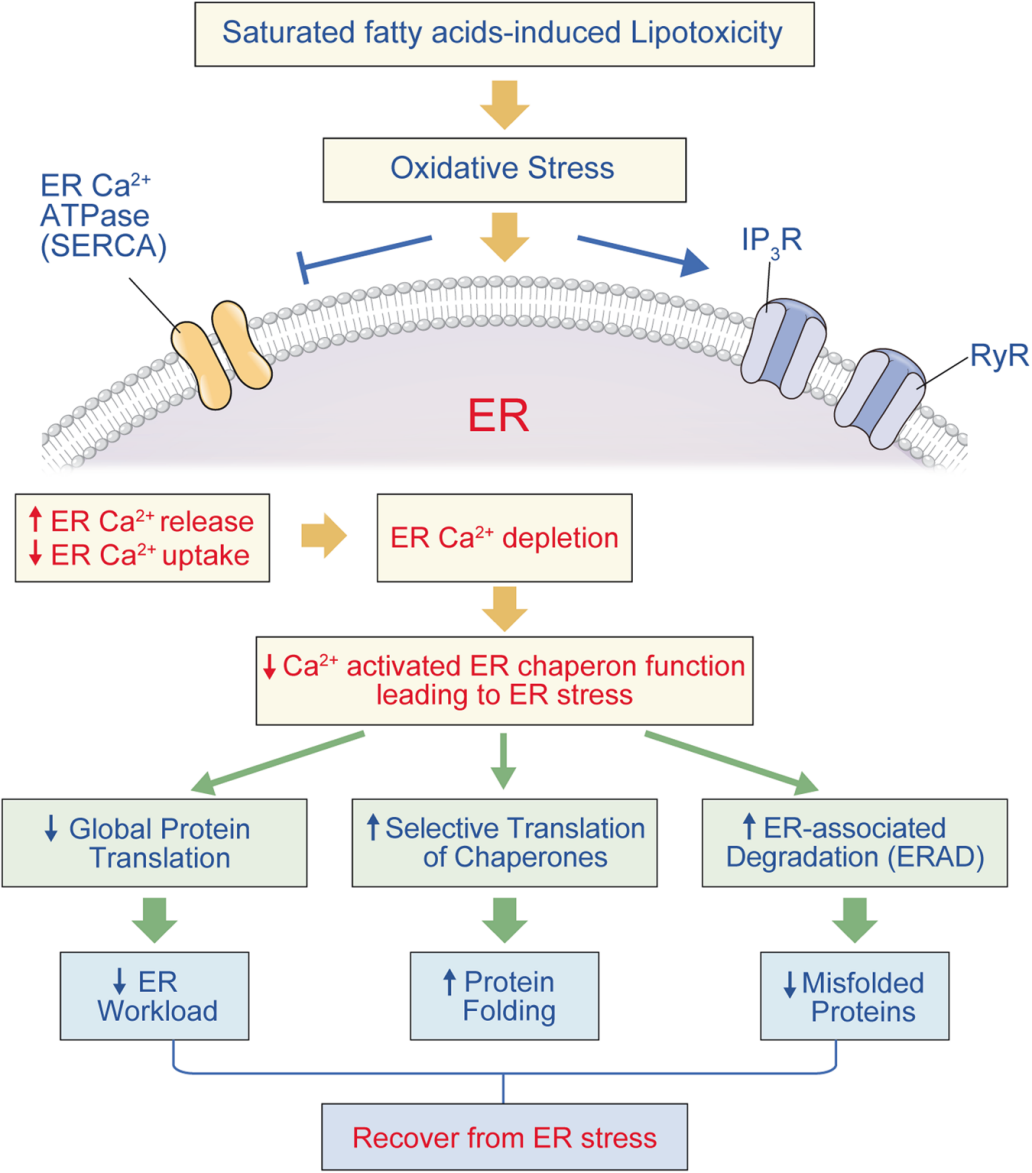
ER Ca^2+^ depletion and stress response in β-cell lipotoxicity. Oxidative stress due to saturated fatty acids accelerates ER Ca^2+^ release and suppresses Ca^2+^ uptake into the ER lumen. Decreased Ca^2+^-activated ER chaperone function resulting from Ca^2+^ depletion induces the ER stress response, consisting of general suppression of protein translation with selective translation of chaperones and accelerated degradation of misfolded proteins in the ER, leading to recovery from ER stress. IP_3_R inositol tris-phosphate receptor, RyR ryanodine receptor.

Another critical role of the ER, in addition to protein folding, is the sensing of cellular stresses and maintaining homeostasis^15^. The accumulation of unfolded or misfolded proteins due to ER dysfunction activates the unfolded protein response (UPR), which attempts to attenuate pathologic progression and recover ER function within a limited range. This process is initiated by three ER membrane proteins: inositol-requiring enzyme 1α (IRE1α), protein kinase RNA-like endoplasmic reticulum kinase (PERK) and activating transcription factor 6 (ATF6). During ER stress, these three distinct signal transduction arms dissociate from 78-kDa glucose-regulated protein (GRP78), also known as binding immunoglobulin (BiP), and activate downstream signaling cascades. Functional consequences of the UPR include (1) the reduction of global protein synthesis by attenuating translation, (2) the promotion of selective translation of chaperones to increase ER protein folding capacity, and (3) signaling for ER-associated protein degradation (ERAD) to eliminate misfolded proteins by the ubiquitin‒proteasome system (UPS) (Fig. 1). However, if the ER is unable to reestablish homeostasis, cell death is initiated through proapoptotic signaling.

Due to active Ca^2+^ transport via SERCA, the luminal Ca^2+^ concentration of the ER remains high (100~800 μM)^16^. This phenomenon is important for the function of chaperones in the ER; thus, depleted ER Ca^2+^ levels cause accumulation of unfolded or misfolded proteins. In addition to pathologies of the ER itself, ER Ca^2+^ release disrupts cytosolic as well as organellar Ca^2+^ homeostasis, including that of mitochondria and lysosomes. Mitochondrial Ca^2+^ overload may be related to superoxide generation and mitochondrial dysfunction, consequently triggering the apoptotic process. The connection between oxidative stress and organellar Ca^2+^ homeostasis by lipotoxicity has been supported by the formation of mitochondria-associated ER membranes (MAMs) in metabolic stress. Notably, a noncanonical function of IRE1 is the regulation of the expression of the MAM protein IP_3_ receptor^17^. This spatial proximity facilitates aberrant Ca^2+^ transfer between the ER and mitochondria, establishing a vicious loop of organelle dysfunction. Increased MAMs have been described in palmitate-treated insulin-secreting cells, and the mechanism involves upregulation of the MAM protein GRP75^18^. Disturbances in lysosomal Ca^2+^ regulation related to ER Ca^2+^ release can impair lysosomal protein degradation.

Intriguingly, unresolved ER stress induces ERAD with additional activation of autophagy, which plays a physiologic protective role against pathological burdens. During the UPR, IRE1α dissociates from BiP/GRP78 and activates c-Jun N-terminal kinases (JNKs), leading to the release of Beclin1 and enhanced basal autophagy^19^. Stimulation of PERK induces ATF4 and CHOP, which drive the expression of autophagy-related proteins, including Atg5 and Atg12, initiating the formation of autophagosomes^20^. However, chronic ER stress blocks autophagic initiation and degradation, which aggravates lipotoxicity in β-cells. Continuous and excessive demand for insulin secretion due to insulin resistance or inefficient compensatory UPR leads to autophagic defects and β cell failure.

## Autophagy defects in beta-cell lipotoxicity

### Role of autophagy in β-cell function

Autophagy is an evolutionarily conserved self-defense process that removes toxic materials and damaged organelles to maintain cellular homeostasis^21^. Over many years, this process has been demonstrated to be critical in cellular physiology and stress defense in mammalian tissues. Autophagy can be classified, with respect to cargo delivery mode and biological functions, into several major categories: macroautophagy, microautophagy, and chaperone-mediated autophagy (CMA). In macroautophagy and related processes (hereafter referred to as autophagy), the major lysosomal degradation pathway is used to eliminate long-lived proteins and intracellular organelles. The autophagic process occurs through a series of steps: initiation, elongation, maturation, fusion, and degradation^22^. The onset of autophagy is marked by the formation of an isolated double membrane (phagophore) surrounding cytoplasmic cargos. The complete engulfment of the material to be degraded by the phagophore forms the autophagosome, which has a vesicle structure that captures the cargos. The autophagosome subsequently fuses with a lysosome to form an autolysosome where material undergoing autophagy is being degraded. Degradation products are recycled back for other cellular processes^23^.

In pancreatic β-cells, autophagy was first described in the context of the intracellular degradation of insulin granules^24,25^. The disposal of aged granules in β-cells is carried out by crinophagy, a process of degrading excess secretory granules containing insulin by delivering them to lysosomes. Crinophagy is required for maintaining insulin granule pools at an optimal level^26^. If any abnormalities exist in the degradation pathway, the imbalance between proinsulin biosynthesis and insulin secretion leads to β-cell dysfunction. Autophagy in β-cells received additional attention after reports about large aggregates of ubiquitinated proteins in insulin-positive β-cells from Zucker diabetic fatty rats^27^. The degradation of these proteins was performed by lysosomes, rather than proteasomes, following the process of autophagy.

Does autophagy have a protective or a detrimental function in β-cells in response to stress conditions? Autophagy defects in a mouse model lacking Atg7 in β-cells (Atg7^f/f^:RIP-Cre mice) resulted in progressive β-cell loss and impaired glucose tolerance^28,29^. This phenotype suggests that autophagy is fundamental for β-cell survival. In addition, autophagy is indispensable for various physiological processes in β-cells, including differentiation, development, and insulin homeostasis^30,31^. Autophagy is also needed to remove dysfunctional mitochondria (mitophagy) to maintain a healthy mitochondrial network through mitochondrial fission and fusion^32^. The ER, as the crucial organelle responsible for insulin biosynthesis, also has an intimate link to autophagy^33^. During the unfolded protein response (UPR), severely damaged fragments become selectively eliminated by autophagy to maintain ER homeostasis, called ER-phagy^34^. Impaired autophagic flux renders the quality control system inefficient and serves to eliminate damaged organelles. This process explains the accumulation of swollen mitochondria and expanded ER in Atg7 knockout β-cells^28,29^.

**Fig. 2:**
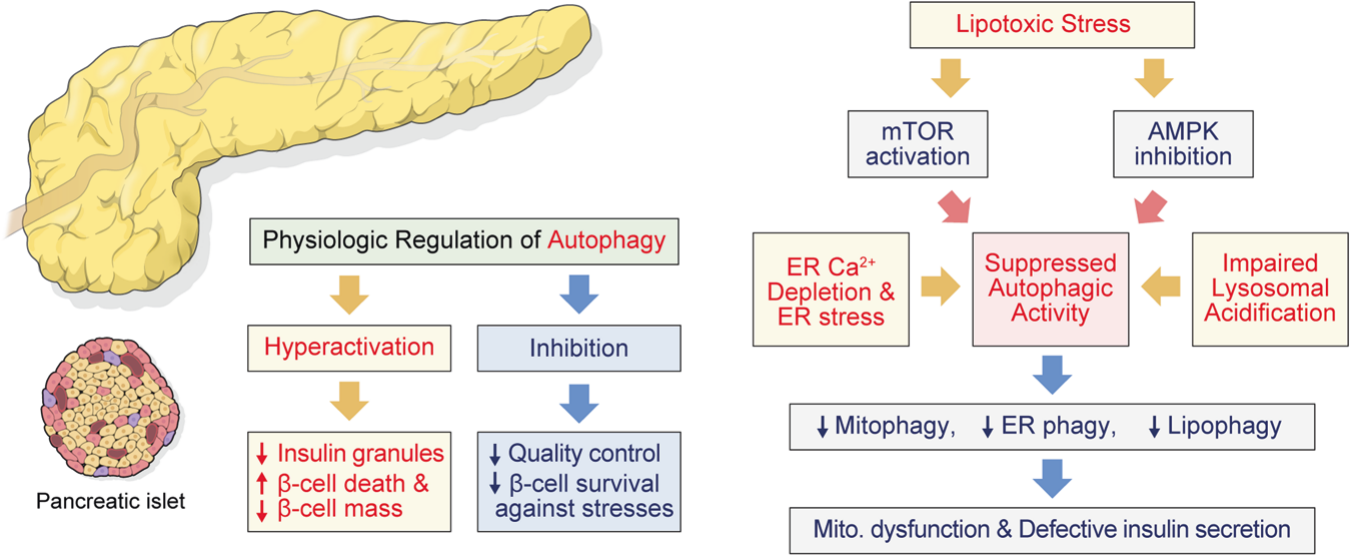
Autophagic defects in β-cell lipotoxicity. Both hyperactivation and inhibition of autophagy reduce the cellular functions and survival of pancreatic islets. Activation of mTOR signaling by AMPK inhibition in β-cell lipotoxicity suppresses the activities of mitophagy, ER-phagy, and lipophagy, causing mitochondrial dysfunction, ER stress, and defective insulin secretion.

While autophagy is protective in β-cells, hyperactivation of autophagy reduces β-cell function and survival both in vitro and in vivo^35^. For example, strong activation of autophagy by rapamycin, as a suppressor of mechanistic target of rapamycin (mTOR), decreases insulin production and exacerbates β-cell death. The autophagy inhibitor 3-methyladenine abrogates the effects of rapamycin and restores insulin secretion, suggesting that β-cell dysfunction induced by rapamycin might be mediated through the excessive induction of autophagy. A similar phenotype has been reported in a Raptor knockout model, in which the deletion induces mTOR inhibition and enhances autophagy. This mouse exhibited compromised insulin secretion, which was rescued by autophagy inhibition^36^. Consistently, knockdown of Atg5/7 or short-term bafilomycin A1 treatment led to autophagy inhibition and enhanced proinsulin biosynthesis and insulin secretion^31^. Thus, well-regulated autophagy is crucial for maintaining β-cell homeostasis. Stresses disturbing autophagic activity, either inhibition or overactivation, contribute to β-cell dysfunction and the development of diabetes (Fig. 2). A recent study showed that proper autophagic function is also needed for the regulation of glucagon secretion in alpha cells^37,38^. However, α-cells are resistant to lipotoxicity, partially explained by abundant expression of antiapoptotic proteins^39^.

### Defective autophagy as a therapeutic target in lipotoxicity

As described above, several mechanisms have been implicated in lipotoxicity, such as ER stress, mitochondrial dysfunction, and oxidative stress. Recently, defective autophagy has emerged as a focus for a new pathogenic mechanism^40^. Consequently, how can excess fatty acids regulate autophagic activity? Could inhibition of this mechanism be a novel therapeutic strategy to protect against lipotoxicity? Images of human islets treated with nonessential fatty acids showed an accumulation of autophagic vacuoles, increased size and number of autophagosomes, and increased β-cell death^41^. The accumulation of autophagic vesicles was also observed in pancreatic β-cells in a mouse model of tacrolimus-induced diabetes, a side effect of the immunosuppressant drug^42^. An increased number of autophagosomes can result from either stimulated autophagosome formation or slowed degradation. Stimulation of autophagosome formation could be due to an increase in autophagic flux by free fatty acids (FFAs)^43–45^; alternatively, FFAs could inhibit autophagic turnover, consequently leading to aggregation of autophagosomes in β-cells^46,47^. These conflicting interpretations of the impact of FFAs on autophagy may be explained by varying time points chosen to examine autophagic activities and/or the use of different inhibitors to follow autophagy.

Autophagy was reported to be suppressed by lipotoxic conditions due to AMP-activated protein kinase (AMPK) inhibition following mTORC1 activation in different cell types^48–50^. Consistent with these reports, a marked downregulation of autophagy-related proteins (Atg5 and Ag7) was observed in obese mice, contributing to autophagy suppression^51^. Furthermore, a high-fat diet challenge in mice resulted in compromised autophagic activity associated with impaired lysosomal acidification that contributes to lipotoxicity in the kidney^52^. Likewise, lipotoxicity-induced inhibition of autophagy in β-cells was normalized by lysosomal acidification, which also restored mitochondrial function^53^. Moreover, dyshomeostasis of intracellular Ca^2+^ has been proposed to inhibit autophagosome-lysosome fusion^54^. Thus, the application of verapamil, a Ca^2+^ channel blocker, restored autophagic flux in the liver and attenuated inflammation and insulin resistance in obese mice^54^. However, the role of voltage-gated Ca^2+^ entry in the intracellular Ca^2+^ homeostasis of hepatocytes, as nonexcitable cells, identifies a Ca^2+^ channel-independent action of verapamil. Of note, verapamil has been shown to inhibit thioredoxin-interacting protein (TXNIP) and the NOD-like receptor pyrin domain containing-3 (NLRP3) inflammasome in a manner independent of Ca^2+^ channels^55^. Moreover, in the clonal β-cell lines INS-1 or MIN6, verapamil stimulates autophagy^55,56^.

The relationship between lipotoxicity and autophagy is intricate, and the pathophysiological mechanism involves multiple factors (Fig. 2). Understanding the contribution of each factor involved in regulating autophagy is critical for discovering therapeutic treatments for lipotoxicity-related disorders. Notably, dysfunctional autophagy caused by the downregulation of key regulators of the process could be reversed by antidiabetic drugs known to modulate autophagy^41,42,57,58^. Metformin, the most commonly prescribed antidiabetic agent, is known to prevent lipotoxic β-cell apoptosis and restore glucose-stimulated insulin secretion^59^. Metformin enhanced the removal of aggregated autophagic vacuoles in β-cells and AMPK-dependent protection from lipotoxicity^57^. The latter study mainly used 2 mM, a suprapharmacological concentration of metformin, while a 100-fold lower dose was sufficient to restore glucose-stimulated insulin secretion in islets from type 2 diabetic organ donors. The acute restoration of insulin secretion by metformin is caused by inhibition of voltage-dependent anion channel-1 (VDAC1), which is mistargeted to the β-cell plasma membrane in diabetes^60^. Insulin sensitizers, such as thiazolidinediones, have been reported to stimulate autophagy associated with activation of AMPK and suppression of mTORC1^61^.

Exendin-4, a glucagon-like peptide-1 (GLP-1) analog, also protects against β-cell lipotoxicity via a number of mechanisms, including induction of the ER chaperone and antiapoptotic BiP/GRP78 protein^62^, inhibition of proapoptotic stress kinases^63^, and restoration of lysosomal function and autophagic flux^42,64^. Exendin-4 prevented the excessive accumulation of autophagosomes and restored autophagic clearance in defective β-cells^42^. GLP-1 agonists as well as the GLP-1-degrading dipeptidyl peptidase-4 inhibitor also increase LC3 II and autophagosome formation, restoring insulin secretion in beta-cells from high-fat diet obese or diabetic mice^65–67^. However, there was no effect of exendin-4 on autophagy in the absence of glucolipotoxicity^67^.

Sodium-glucose cotransporter 2 (SGLT2) inhibitors are a newly introduced class of antidiabetic drugs that reduce circulating glucose levels through the induction of glycosuria. SGLT2 inhibitors were reported to activate autophagy and preserve normal morphology and function in renal cells or tissues from diabetic mice^68,69^. In a recent study, an SGLT2 inhibitor or knockdown of the SGLT2 transporter restored autophagic levels via AMPK activation and mTOR inhibition in a human proximal tubular cell line cultured in high-glucose medium^70^. The direct molecular mechanisms explaining the beneficial effects of SGLT2 inhibitors in isolated cells are not fully understood but are consistent with the concept that SGLT2 acts as a sensor of excess nutrients. Inhibition of this molecule causes intracellular signaling linked to nutrient deprivation and AMPK-mediated autophagy activation even in cell types or tissues that do not express SGLT2^71^. In addition to currently known antidiabetic drugs, other agents that positively modulate autophagy, such as the antiarrhythmic drug amiodarone, were shown to recover β-cell function in islet amyloid polypeptide-expressing insulinoma and human islet cells^72,73^. These results suggest that modulating autophagy is a promising strategy to counteract beta-cell loss and the development of diabetes.

## Lysosomal calcium regulation and autophagy

### Regulation of lysosomal Ca2+ signaling

Lysosomes are a group of membrane-enclosed organelles containing the bulk of digestive enzymes found in most eukaryotic cells^74^. As lysosomes have more than fifty acid hydrolases, the primary role of lysosomes has been regarded as a degradation system for damaged organelles and cellular macromolecules. Recently, lysosomes have been shown to have many additional functions to maintain cellular homeostasis by engaging with other compartments^75^. Lysosomal signal sensing and degradation have been proposed to cooperate to control fundamental physiological processes^76^. In response to different environmental cues, the lysosomal signaling network functions to induce adaptive responses as well as secure proper cellular demand for degradation. Moreover, the degradative process provides catabolites that act as a nutrient-sensing signal to turn on adaptive responses. Given this important association, it is not surprising that any disturbance of lysosomal degradation and signal sensing leads to the development of pathological conditions, such as lysosomal storage disorders or neurodegenerative diseases.

Lysosomes carry diverse lysosomal membrane proteins which function to transport metabolites, enzymes, and ions across the membrane. The transporting activities generate luminal acidification and are directly involved in regulating proper lysosomal functions. Particularly, by harboring the H^+^ ATPase pump, lysosomes have a unique strongly acidic luminal pH (4.5–5.0) that favors the activity of degradation enzymes. These organelles also contain a wide range of other ion channels that transport H^+^, Na^+^, K^+^, Ca^2+^, and Cl^−^ driven by the electrochemical gradient across the lysosomal membrane^77^. In the past, it was reported that the function of lysosomes was mostly dependent on the activity of proton pumps and luminal acidification, and most studies have focused on pathological changes in lysosomal pH^78^. Currently, this view has changed to include other ion channels with advances in lysosomal patch-clamp technique^79,80^.

**Fig. 3:**
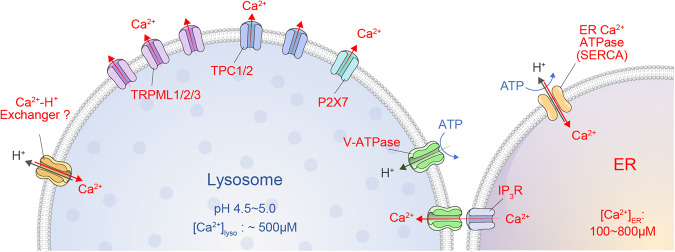
Regulation of lysosomal Ca^2+^ homeostasis. The lysosome contains different classes of Ca^2+^ channels, including transient receptor potential cation channels of the mucolipin family (TRPML1-3), two-pore channels (TPC1-2), and ionotropic purinergic receptors (P2X4). These channels induce Ca^2+^ release into perilysosomal domains driven by high Ca^2+^ gradients, which possibly develop by Ca^2+^ transfer from the ER or transporters' action related to the acidic luminal pH of the lysosome.

Ca^2+^ signaling is critical for lysosomal functions such as lysosomal mobility, degradation, and connection at membrane contact sites. Lysosomes are considered an intracellular Ca^2+^ store, along with the ER, having a high luminal Ca^2+^ concentration of ~ 500 μM, more than 5000-fold higher than cytosolic Ca^2+7^. Ca^2+^ efflux is conducted via at least three types of lysosomal Ca^2+^ channels: transient receptor potential cation channels of the mucolipin family (TRPML1-3), two-pore channels (TPC1-2), and trimeric Ca^2+^ two transmembrane channels (P2X4) (Fig. 3). The uptake of Ca^2+^ into lysosomes is not yet well elucidated. Several studies support the idea that a Ca^2+^/H^+^ exchanger exists due to a notable reduction in lysosomal Ca^2+^ when lysosomal pH is disturbed by a V-ATPase inhibitor or alkalizing reagents such as NH_4_Cl^7,81–83^. However, the ER is proposed to serve as the main source to refill lysosomal Ca^2+^ via IP_3_R^8^. However, the route and mechanism by which Ca^2+^ is transported into lysosomes remain unclear. A putative Ca^2+^ channel that resides on the contact site between the ER and lysosomes has been implicated.

In the history of lysosomal Ca^2+^ studies, nicotinic acid adenine dinucleotide phosphate (NAADP) was the first potent reagent discovered to strongly induce Ca^2+^ release from lysosomes. Initially, NAADP administration provoked a cytosolic Ca^2+^ rise originating from a Ca^2+^ store that is insensitive to IP_3_ and cyclic ADP-ribose^84^. Following this, the source of Ca^2+^ release induced by NAADP was shown to originate from the endolysosomal system^85–87^. With a better understanding of the important role of lysosomal Ca^2+^ signaling, more factors have been found to regulate lysosomal Ca^2+^, including pH, nutrients, stressful conditions, or small molecules such as ATP, phospholipids and sphingosine^7,88–91^. Each stimulus modulates lysosomal Ca^2+^ via different mechanisms to trigger selective Ca^2+^ signaling responses that favor the needs of the cell.

In addition, lysosomal signaling was shown to be regulated via contact sites with other membrane-bound organelles, such as the ER, Golgi, mitochondria, and peroxisomes. Many important functional events occur at lysosome-organelle contact sites, including lipid transfer, lysosomal positioning and trafficking. Regarding the regulation of lysosomal Ca^2+^, there might be Ca^2+^-mediated functional coupling at the microdomain between the ER and lysosomes. Indeed, NAADP triggers Ca^2+^ release via TPCs, which requires intact function of IP_3_Rs and RyRs on the ER. This observation suggested a trigger hypothesis by NAADP in which the Ca^2+^ mobilized from lysosomes can initiate a global Ca^2+^ increase via Ca^2+^-induced Ca^2+^ release from the ER^87,92,93^. The triggering function of Ca^2+^ released from lysosomes is nicely illustrated in adrenaline-induced glucagon secretion from pancreatic α-cells, which is attenuated in TPC-2 channel KO mice^94^, while such channel deletion does not alter insulin secretion^95^. In addition, a membrane contact site between mitochondria and lysosomes was identified to facilitate the direct transfer of Ca^2+^ from lysosomes to mitochondria^96^. Lysosomal TRPML1-mediated Ca^2+^ efflux was shown to transfer Ca^2+^ into mitochondria via VDAC1 and MCU. This new discovery contributes an additional mechanism for regulating Ca^2+^ dynamics, which might be implicated in pathological diseases, including neurodegenerative and lysosomal storage disorders^96–98^.

### Autophagy regulation by lysosomal Ca^2+^

Lysosomal Ca^2+^ is one of the essential factors needed for various physiological processes, including endocytic membrane trafficking, autophagy, membrane repair, formation of ER-lysosomal contact sites, and protein transport^75^. Most studies have revealed the important role of TRPMLs, and some have highlighted TPCs and P2X4 in autophagy and lysosomal system regulation. TRPMLs belong to a large family of transient receptor potential ion channels containing three isoforms (TRPML1, 2, and 3). TRPML1 mainly localizes in lysosomes, while TRPML2 and TRPML3 are found to reside on early endosomes, late endosomes and lysosomes^99^.

TRPML1 is the best-studied channel regarding lysosomal adaptation and autophagy regulation. The first report about the role of TRPML1 in pathophysiology characterized a disorder affecting the lysosomal pathway, so-called mucolipidosis type IV (MLIV)^100^. The TRPML1-mutated cells clearly show defective autophagic processes with impaired lysosomal pH, enlargement of lysosomes and autophagosomes along with accumulation of undigested materials. The pathogenic manifestations of TRPML1 mutation include the increased formation of new autophagosomes and a delay in autophagosome-lysosome fusion^101^. Imaging studies revealed substantial aggregation of cytoplasmic bodies in the cerebral cortex of TRPML1 knockout mice. As a result of suppressed autophagic degradation, the levels of LC3-II and p62 were markedly increased, suggesting that macroautophagy is defective in mucolipin-1-deficient neurons. This observation, together with the characteristics of MLIV fibroblasts, contributes new insight into the neuronal pathogenesis of this disease^102^.

In fasting conditions, as a molecular mechanism of autophagic regulation, lysosomal Ca^2+^ release via TRPML1 was enhanced, generating a high Ca^2+^ microdomain surrounding lysosomes^103^. The increased Ca^2+^ level in the perilysosomal microdomain activates the calcium-dependent serine/threonine phosphatase calcineurin and dephosphorylates transcription factor EB (TFEB), enabling the translocation of TFEB into the nucleus, where it activates the transcription of lysosomal biogenesis and autophagy-related genes. Another study proposed TRPML1-dependent autophagy activation by reactive oxygen species (ROS)^88^. In response to ROS generation, TRPML1 is directly activated and releases Ca^2+^, followed by calcineurin stimulation and TFEB translocation into the nucleus. Genetic or pharmacological intervention to suppress TRPML1 prevents the removal of damaged and ROS-generating mitochondria. Thus, TRPML1 is a positive regulator of autophagy required for autophagosome-lysosome fusion and transcriptional upregulation of autophagy-related genes.

TRPML2 and TRPML3 have received less attention regarding autophagy regulation. Nevertheless, TRPML3 may play an important function as a regulator of membrane trafficking and autophagy^104^. Overexpression of TRPML3 enhances autophagy, and knockdown or loss-of-function mutation of TRPML3 was shown to inhibit autophagy. TRPML3 was reported to specifically bind to GATE16, a mammalian Atg8 homolog, to facilitate autophagosome maturation by providing Ca^2+^ during the membrane fusion process^105^. Each TRPML channel plays a distinct role in autophagy, as noted earlier. Furthermore, the role of TRPML heteromultimerization was proven to regulate starvation-induced autophagy and cell viability, indicating that hetero-TRPMLs, not a distinct type, might be more important in the context of regulating autophagy^106^.

In addition to TRPMLs, another lysosomal Ca^2+^ flux regulator of autophagy is the TPC. Mammalian cells contain two forms of TPCs: TPC1 resides on endosomes and lysosomes, and TPC2 localizes to lysosomes^83,107^. Due to the specific localization of TPCs on the lysosomal membrane and their regulation of Ca^2+^ efflux, TPCs were predicted to be involved in autophagy regulation. However, it remains controversial whether TPCs act as enhancers or inhibitors of autophagy. Several studies have proposed that Ca^2+^ signaling from TPCs is needed for the activation of autophagy. NAADP, a well-known agonist of TPCs, triggers Ca^2+^ release and regulates the autophagic process in astrocytes by increasing lysosome formation and two autophagic markers, LC3-II and Beclin1^108^. The Ca^2+^ signal evoked by NAADP is linked to calcium/calmodulin-dependent protein kinase kinase 2 (CaMKK2) and AMPK to promote autophagosome formation^109^. This Ca^2+^-dependent signaling also influences autophagic degradation at the lysosomal level. The link between TPCs and autophagy was postulated clearly in TPC knockout mice^110^. In the absence of TPC activity, autophagic flux was decreased in cardiomyocytes upon starvation, suggesting the critical role of TPCs in appropriate basal and induced autophagic flux in cardiac tissues^110,111^.

Conversely, TPCs have been considered a negative regulator of autophagy via effects on lysosomal pH^112^. Lu et al. reported that TPC2 overexpression or activation of TPC2 by NAADP inhibited autophagosome-lysosome fusion. However, knockdown of TPC2 or application of a TPC2 antagonist (Ned-19) reduced TPC2-dependent autophagosome accumulation. The molecular mechanism demonstrated that TPC2/NAADP/Ca^2+^ signaling alkalinizes lysosomal pH to specifically inhibit the later stage of basal autophagic progression. In another knockout experiment, TPC2^−/−^ mice exhibited an atrophic phenotype with enhanced autophagic flux under starvation, which was different from the study mentioned above^112^. This discrepancy was thought to result from different strategies used in generating the TPC2 knockout mice. As a lysosomal Ca^2+^ channel, P2X4R (purinergic receptor P2X4) participates in the fusion step of autophagy by regulating Ca^2+^ release. A complex formed by the interaction between P2X4 and calmodulin (CaM) at the endolysosomal membrane promotes fusion and vacuolation in a Ca^2+^-dependent fashion^113^.

## Role of mTOR in autophagy regulation

### Regulation of mTOR signaling at the lysosome

**Fig. 4:**
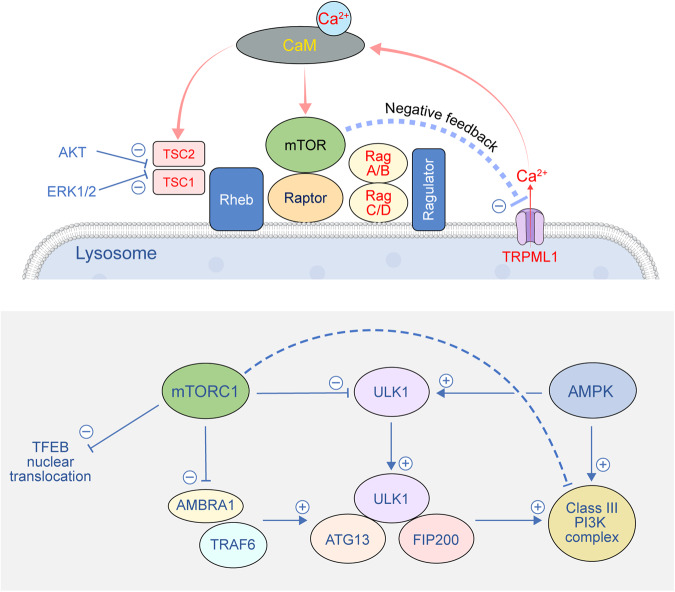
Role of mTOR signaling in autophagy regulation at lysosomes. During activation of mTOR signaling, Rheb and Ragulator induce the localization of mTOR on the lysosomal surface. Autophagic processes mediated by Unc-51-Like Kinase 1 (ULK1) and the phosphatidylinositol 3-kinase (PI3K) complex are negatively regulated by mTORC1 signaling on the lysosome. Perilysosomal Ca^2+^ release via the TRPML1 channel is critically involved in mTOR activation by promoting interaction with calmodulin (CaM), which in turn causes negative feedback regulation by suppressing TRPML1 activity. FIP200 FAK family kinase-interacting protein of 200 kDa, Rheb Ras homolog enriched in brain, TFEB transcription factor EB, TSC Tuberous sclerosis complex, TRAF6 TNF receptor-associated factor 6.

The mTOR complex, a master regulator of cell growth, has two major targets of rapamycin: mTOR1 and mTOR2, which not only have distinct roles but also regulate each other to maintain growth and proliferation^114^. mTORC1 functions as a main mediator of protein synthesis and cell growth, whereas mTORC2 is involved in regulating cell proliferation in response to growth factors as well as metabolism. A canonical signaling cascade to activate mTORC1 requires both active Rags and GTP-bound Ras homolog enriched in brain (Rheb) residing on the lysosomal surface. Rag-GTPases, members of the Ras family of GTP-binding proteins, regulate mTORC1 in an amino acid-dependent manner (Fig. 4). In response to increased amino acid abundance, Rag heterodimers interact with the Raptor component of mTORC1, which induces the redistribution of mTORC1 to Rab7-containing lysosomes^115^. The movement of mTORC1 to the lysosomal surface is driven by the interaction between the trimeric Ragulator protein complex of mTORC1 and Rag GTPases^116^. After translocating to the lysosomal surface, mTORC1 interacts with its kinase activator, Rheb GTPase, to enhance the binding of 4E-BP1 to mTORC1. Under conditions of limited amino acids, Rap1-GTPases concentrate lysosomes in the perinuclear area and reduce the available lysosomal surface for mTORC1 activation^117^. The absence of Rap1 expands the lysosome population, increasing the association between mTORC1 and activators on lysosomes, resulting in mTORC1 activation.

In addition to canonical pathways regulating mTORC1, different extracellular and intracellular signal inputs have been reported to control mTORC1 activity. Notably, the intracellular second messenger Ca^2+^ has been shown to activate the p70 ribosomal S6 kinase, which is situated directly downstream of mTORC1, as shown in rat liver epithelial cell lines^118–120^. The activating mechanism of mTORC1 by amino acids has also been demonstrated to be Ca^2+^-dependent^121^. Amino acids increase cytosolic Ca^2+^ binding to CaM, in turn activating hVPS34 and mTORC1 signaling. The mTOR signal was diminished by intracellular Ca^2+^ chelators, a CaM antagonist, or knockdown of CaM, suggesting that intracellular Ca^2+^ and CaM are involved in amino acid-induced mTORC1 activation^122^. Another mechanism has been suggested for amino acid activation of mTORC1 through regulation of the tuberous sclerosis complex 2 (TSC2)-Rheb axis by Ca^2+^/CaM^123^. These interesting findings prompted a surge of questions as to which intracellular Ca^2+^ source directly plays the critical role in mediating mTORC1 activation. Several studies have reported that lysosomal Ca^2+^ dyshomeostasis observed in lysosomal storage diseases might be responsible for the inhibition of the mTORC1 signaling pathway. Indeed, in flies lacking TRPML1 function, mTORC1 signaling was attenuated. Reactivation of mTORC1 by a high-protein diet reduced the severity of the mutant TRPML1 phenotype, indicating the interrelationship between the TRPML and TORC1 pathways^124,125^. Consistent with these findings, TRPML1-mediated lysosomal Ca^2+^ release activates mTORC1 by promoting interaction with CaM^122^. Furthermore, negative feedback regulation of mTORC1 activity on TRPML1 through a CaM-dependent mechanism has been shown to be important for maintaining cellular homeostasis during starvation^126^.

### mTOR activation in β-cell lipotoxicity

With a full nutrient supply, mTORC1 is rapidly activated to promote biosynthesis, which generates new cellular materials such as proteins, lipids, and nucleic acids. In contrast, during starvation, an adaptive mechanism is turned on to suppress mTORC1 activity to conserve the limited cellular energy and enhance the production of recycled materials via the degradation pathway. Depending on the cell type, mTORC1 plays a tissue-specific role in contributing to the maintenance of energy homeostasis^127–129^. In pancreatic β-cells, mTORC1 has been implicated in cell proliferation by modulating critical factors of the cell cycle, including cyclin D2, cyclin D3 and Cdk4, in response to growth factors, insulin and nutrients^130^. In addition, the importance of mTORC1 activity in β-cells has been documented by the integration of several growth signaling pathways, such as protein kinase B (AKT), protein kinase C, Hippo, epidermal growth factor receptor, and synapses of amphids defective protein kinase A^130–134^. In a genetic approach, gain or loss of mTORC1 function provided important insights into the role of mTORC1 in β-cell physiology. Knockout of Raptor, a core component of mTORC1, in mouse β-cells resulted in a diabetic phenotype, impaired glucose-stimulated insulin secretion (GSIS) and decreased β-cell viability and proliferation. The underlying mechanisms revealed that the 4E-BPs/eIF4E arm of mTORC1 regulates β-cell proliferation, while S6K controls cell size, autophagy and apoptosis. In addition, the mTORC1/4EBP2/eIF4E pathway is implicated in insulin processing via cap-dependent translation of carboxypeptidase E^135^. Hyperactivation of mTORC1 by overexpressing Rheb resulted in an increase in β-cell mass and insulin secretion^136^. Furthermore, inhibition of mTORC1 by rapamycin has been shown to trigger the onset of diabetes^137^. Intriguingly, another study showed biphasic effects of mTORC1 overactivation by the deletion of TSC2: β-cell mass as well as an increase in insulin secretion were increased in young (up to 30 weeks), while hyperglycemia developed along with insulin resistance due to β-cell exhaustion after 40 weeks of age^138^. This result indicates that prolonged and continuous upregulation of mTORC1 exerts deleterious effects and causes β-cell pathology in contrast to its physiologic roles. Upon chronic exposure to excess nutrients, mTORC1 in β-cells is consistently activated, which is accompanied by an increase in β-cell death. Notably, relative to that of the controls, mTORC1 was upregulated in islets from organ donors with type 2 diabetes, while mTORC2 was downregulated^139^. Consistently, genetic and pharmacological suppression of mTORC1-S6K1 signaling recovered insulin secretion in diabetic patient islets. The same results were observed in islets exposed to glucotoxic conditions and in islets from diabetic mice.

How does the sustained activation of mTORC1 cause β-cell dysfunction? Constitutively active mTORC1 has a negative impact on downstream signaling, including mTORC1-S6K1-IRS and mTORC2, as well as other intracellular processes, such as ER homeostasis and autophagy^140^. There are negative feedback loops between mTORC1-S6K1 and the mTORC2-AKT axis that prevent excessive insulin downstream signaling cascades. Hyperactivation of mTORC1 can downregulate RTK-IRS1/2-PI3K-AKT signaling. Another suggested mechanism is the suppression of autophagy by mTORC1. Early observations using electron microscopy showed the abnormal accumulation of autophagosomes in β-cells after exposure to high fatty acid and glucose or in islets of T2D patients^41,141^. Further studies reported that autophagic flux was blocked by a high-fat diet or under excessive nutrient conditions^141,142^. In this context, inhibition of mTORC1 was shown to improve autophagic activity and β-cell survival against overnutrition stress. One of the molecular mechanisms was demonstrated to be mTORC1-mediated Unc-51-Like Kinase 1 (ULK1) phosphorylation at Ser757, which prevented autophagy initiation^143^. Conversely, AMPK promotes autophagy by directly activating ULK1 through phosphorylation of Ser317 and Ser777. Along with autophagic suppression under overnutrition stress, prolonged mTORC1 upregulation induces ER stress and apoptosis in human and rodent islets and clonal β-cells^141,144,145^. Accordingly, hyperactivation of mTORC1 in TSC2-KO mice resulted in strong induction of UPR markers, including PERK, p-eIF2α, ATF4 and CHOP.

### Autophagy regulation by mTORC1

Given the important role of autophagy, the multiple signaling modalities involved in the regulation of the process have been divided into two main categories: mTOR-dependent and mTOR-independent pathways^146^. The first study on autophagy regulation by mTOR reported the control mechanism of Tor (a homolog of mTOR) for autophagy induction in yeast, which blocks a factor required for autophagy initiation^147^. Several groups have proposed that mTORC1 is regulated by phosphorylating the autophagy regulatory complex formed by ULK1-Atg13-FIP200, which subsequently inhibits autophagy initiation^148–150^. In addition, AMBRA1, interacting with the E3 ligase TNF receptor-associated factor 6 (TRAF6), leads to Lys63 ubiquitylation and stabilization of ULK1, which enhances its kinase activity and autophagy induction (Fig. 4). However, under nonautophagic conditions, mTORC1 inhibits AMBRA1 by phosphorylation and subsequently inhibits autophagy^151^. Another mechanism has been suggested according to which mTORC1 specifically inhibits the ATG14-containing autophagic class III phosphatidylinositol 3-kinase (PI3K) complex, which is involved in autophagy induction through phosphorylation of ATG14 at multiple sites^152^. mTORC1 has also been implicated in autophagy inhibition by the phosphorylation of NRBF2/Atg38, which has been identified as the fifth subunit of the autophagic class III phosphatidylinositol 3-kinase complex^153^.

Recently, mTORC1 has been shown to not only participate in the early step of autophagy but also extensively function at later stages, including autophagy elongation and maturation (summary in Table 1). The elongation step is one of three major steps in autophagy that results in complete autophagosome formation. mTORC1 has been shown to be involved in this step via the phosphorylation of WIPI2 at Ser395, a critical protein in the growth of the isolation membrane and elongation, promoting the interaction between WIPI2 and the E3 ubiquitin ligase HUWE1 for ubiquitination and proteasomal degradation^154^. Additionally, mTORC1 was shown to inhibit autophagy via p300 phosphorylation, which reduces the acetylation of several autophagy-related proteins such as LC3, Atg5, and Atg7^155^. Regarding the late stage of autophagy, mTOR regulates factors participating in autophagosome-lysosome fusion, including UV radiation resistance-associated gene (UVRAG) and proteins associated with UVRAG as an autophagy enhancer (Pacer)^156,157^. mTORC1 interacts with and phosphorylates UVRAG, which prevents the interaction with the HOPS complex, a component of the late endosome/lysosome fusion machinery, enhancing autophagosome and endosome maturation^156^. In response to nutrients, mTORC1 phosphorylates Pacer at serine 157 to disrupt its association with Stx17 and the HOPS complex, thus preventing Pacer-mediated autophagosome maturation^157^. The function of mTORC1 in autophagy is not only via post-translational modification but also through the transcription factors TFEB, TFE3 and microphthalmia-associated transcription factor (MITF) to regulate lysosome biogenesis and autophagy. TFEB and TFE3 were identified as transcription factors controlling genes involved in autophagosome formation, fusion of autophagosomes with lysosomes, and lysosomal biogenesis^158,159^. The activity of mTORC1 negatively regulates autophagy by phosphorylating different serine residues in TFEB/TFE3 (Ser211, Ser122, Ser142 on TFEB, and Ser321 on TFE3), which promotes the binding between TFEB/TFE3 and the 14-3-3 family of proteins, resulting in their retention in the cytosol^160–163^. Therefore, the two master regulators can no longer turn on lysosomal gene expression, which has negative impacts on autophagy. Another member of the MiT/TFE family, MITF, was able to induce autophagy via upregulation of microRNA 211 (miR211)^164^. Inhibition of mTORC1 by the Torin-1 compound induces nuclear translocation of MITF and triggers the expression of genes involved in autophagy.

**Table 1:** mTOR-mediated regulation of autophagic processes.

Signaling		Phosphorylation action of mTOR	Reference
Autophagy induction	ULK-ATG13L-FIP200 (ATG1-ATG13-ATG17 in yeast)	- ATG13L (Ser258) and ULK1 (Ser637): Suppresses ULK1 kinase activity	^148–150^
		- ULK1 (Ser757): Disrupts the interaction between ULK1 and AMPK	^143,204^
	PIK3C3/VPS34 complexes	- ATG14 (Ser3, Ser223, Thr233, Ser383, Ser440): Inhibits PIK3C3 kinase activity of ATG14-containing PIK3C3	^152^
	AMBRA1	- AMBRA1 (Ser52): Inhibits the interaction with E3-ligase TRAF6, which stabilizes ULK1 self-association	^151^
	NRBF2	- NRBF2 (Ser113, Ser120): Blocks Ptdlns3K lipid kinase activity and autophagic process.	^153^
Autophagy elongation	WIPI2	- WIPI2 (Ser395): Prevents autophagosome formation by directing WIPI2 to ubiquitination	^154^
	p300	- P300 (Ser2271, Ser2279, Ser2291, Ser2375): Disrupts intramolecular inhibition of p300, which in turn decreases acetylation of LC3 and loss of the LC3-Atg7 interaction	^155^
Autophagy Maturation	UVRAG	- UVRAG (Ser498): Positively regulates the association of UVRAG with RUBICON, thereby inhibiting UVRAG-mediated autophagosome maturation	^156^
		- UVRAG (Ser550, Ser571): Activates UVRAG/VPS34 leading to decreased lysosomal tubulation upon prolonged starvation	^205^
	Pacer	- Pacer (Ser157): Disrupts the association of Pacer with Stx17 and HOPS complex, which inhibits autophagosome maturation	^157^
	TFEB/TFE3	- Phosphorylates TFEB (Ser211, Ser122, Ser142) and TFE3 (Ser321) to promote the binding of TFEB and 14-3-3 protein and retention in the cytosol.	^160–162,206,207^
		- Phosphorylates TFEB (Ser138 and Ser142) to facilitate the nuclear export of TFEB	^163,208^
	MITF	- mTORC1 inhibition induces MITF translocation, which enhances lysosomal biogenesis	^164^

By functioning at different autophagic steps, mTORC1 is recognized as the master regulator of autophagy. Under nutrient-rich conditions, mTORC1 is known to inhibit the process at various steps of autophagy. Intriguingly, autophagy and mTORC1 have been shown to reciprocally regulate each other. During starvation, mTORC1 is inhibited, which is needed for the induction of autophagy. However, prolonged starvation leads to the reactivation of mTORC1 to restore the lysosomal system to maintain homeostasis^165^. Thus, there is an evolutionary cycle between autophagy and the master regulator mTORC1 to maintain the balance of the intracellular system, so any factor disrupting the regulatory cycle would lead to pathologic conditions and eventually the development of diseases.

## Role of AMPK in autophagy regulation

### Activation of AMPK signaling

**Fig. 5:**
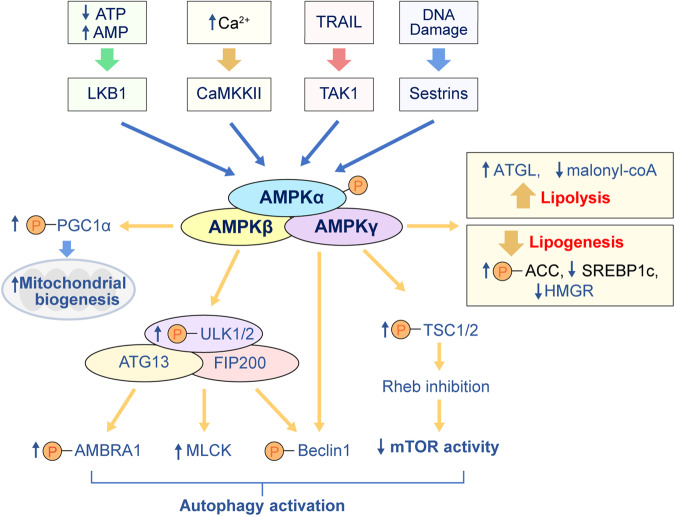
Upstream and downstream regulators of AMPK signaling related to autophagy and metabolism. Lowered ATP, increased Ca^2+^, TNF-related apoptosis-inducing ligand (TRAIL), and DNA damage can trigger AMPK activation mediated by liver kinase B1 (LKB1), calcium/calmodulin (CaM)-dependent protein kinase kinase 2 (CaMKKII), transforming growth factor-β activating kinase (TAK1), and sestrins, respectively. AMPK promotes autophagy by directly activating ULK1 and beclin1 as well as suppressing mTOR activity. AMPK increases mitochondrial biogenesis with activation of proliferator-activated receptor-γ coactivator 1α (PGC1α). AMPK decreases lipogenesis and cholesterol synthesis by inhibiting acetyl-CoA carboxylase (ACC) and HMG-CoA reductase (HMGR). Conversely, AMPK activates adipose triglyceride lipase (ATGL) for lipolysis and facilitates mitochondrial fatty acid uptake for β-oxidation by reducing an allosteric inhibitor, malonyl-CoA. MLCK Myosin light-chain kinase.

AMPK, similar to mTOR signaling, is also an evolutionarily conserved key sensor and master regulator of metabolism. Under physiological or pathological conditions such as exercise, starvation, hypoxia, and shock, AMPK is activated through phosphorylation of upstream kinases related to a high AMP/ATP ratio or Ca^2+^ signaling. Activation of AMPK promotes catabolic processes such as glycolysis, fatty acid oxidation, and autophagic degradation and suppresses anabolic processes including synthesis of proteins, fatty acids, or cholesterol^166^. Mammalian AMPK is a heterotrimeric complex composed of three subunits: an α subunit harboring a protein kinase catalytic domain and noncatalytic β and γ regulatory subunits (Fig. 5). A serine/threonine kinase domain exists in the amino-terminal region of AMPKα, which contains the activation loop, playing a pivotal role in its regulation. In particular, phosphorylation of Thr172 in the activation loop is needed for maximal activities of AMPK^167^.

Three upstream kinases have been known to phosphorylate Thr172 in AMPK: liver kinase B1 (LKB1), calcium/CaM-dependent protein kinase kinase 2 (CaMKK2 or CaMKKβ), and transforming growth factor-β activating kinase (TAK1)^168–170^. First, the tumor suppressor LKB1 is a serine/threonine kinase functioning as a heterotrimer with two other subunits, the STE20-related adapter protein (STRAD) and the scaffolding mouse protein 25 (MO25)^171^. Genetic deletion of *Lkb1* abrogates the activation of AMPK by agonists such as aminoimidazole-4-carboxamide ribonucleoside (AICAR), metformin or phenformin or in response to energy stress, revealing that LKB1 is responsible for the majority of AMPK activation^172^. Increases in AMP or ADP activate AMPK mainly by promoting the phosphorylation of LKB1 but also by inhibiting dephosphorylation by protein phosphatases or directly activating AMPK^173^.

Second, activation of CaMKK2 by intracellular Ca^2+^ phosphorylates Thr172 in AMPK, thus linking Ca^2+^ signaling to the regulation of energy metabolism^169^. Upstream signals for Ca^2+^ elevation, such as adiponectin receptor activation or muscle contraction (exercise), can increase Ca^2+^-bound CaM and activate CaMKK2 and AMPK^174^. As previously mentioned, the ER maintains cellular Ca^2+^ homeostasis and generates signals by releasing Ca^2+^. A relationship between AMPK and the ER-localized protein stromal interaction molecule 2 (STIM2) has been demonstrated^175^. The interaction among STIM2, CaMKK2, and AMPK could be promoted by the release of ER Ca^2+^ and store-operated Ca^2+^ entry.

TAK1 is a serine/threonine protein kinase of the mitogen-activated protein kinase (MAPKK) family, proposed as an alternative third AMPK kinase. TAK1 is induced in response to TNF-related apoptosis-inducing ligand (TRAIL), resulting in a cytoprotective autophagic response mediated by AMPK^176^. Stress-inducible proteins, sestrins, were identified to mediate DNA damage-induced AMPK activation. Sestrin1/2 induces AMPK phosphorylation at T172 and enhances AMPK-mediated mTOR suppression^177^.

The lysosome is known as a center of amino acid sensing and regulation of mTOR signaling^178^. Recently, the lysosome has emerged as a regulatory and functional site for AMPK, promoting the idea of lysosomes as hubs of cellular metabolic regulation^179^. Nutrient deprivation, such as glucose deprivation, which is not associated with gross changes in cellular ATP levels, promotes the formation of an LKB1-AMPK complex at the lysosomal surface^180^. AMPK not only localizes to the endolysosomal compartment but also mediates lysosomal biogenesis by regulating the nuclear translocation of TFEB/TFE3^181^. The target genes for TFEB/TFE3 carry a common genetic motif, coordinated lysosomal expression and regulation (CLEAR), which induces the expression of a network of lysosomal hydrolases, lysosomal membrane proteins, and autophagy-related proteins in response to pathways sensing lysosomal stress^158^. TEFB/TFE3 also induces TRPML1, which can regulate lysosomal membrane fusion with the plasma membrane, eliciting lysosomal exocytosis or autophagosome formation of autophagolysosomes and autophagic degradation. In addition to transcriptional upregulation of autophagy-related proteins, AMPK activation is involved in the initiation, maturation, and processing of autophagy in the endolysosome system.

### Regulatory actions of AMPK on metabolism and autophagy

Upon energetic crisis, AMPK reprograms metabolic activity by covalent modifications, transcriptional regulation, altered substrate utilization, and ultimate restoration of cellular and whole organismal homeostasis^182^. AMPK reduces lipid storage and promotes mitochondrial fatty acid oxidation. The downstream enzymes inhibited by AMPK include mTOR, acetyl-CoA carboxylase (ACC), HMG-CoA reductase (HMGR), and fatty acid synthase (Fig. 5). AMPK phosphorylates and inhibits ACC, the enzyme catalyzing the conversion of acetyl-CoA into malonyl-CoA, the first step in fatty acid synthesis. AMPK also inhibits HMGR, which is the rate-limiting enzyme of cholesterol synthesis^182^. The phosphorylation of SREBP1c and SREBP2 by AMPK inhibits their activities, but there are additional indirect mechanisms involving the regulation of SREBP-mediated lipid synthesis by AMPK. AMPK activates adipose triglyceride lipase (ATGL), catalyzing lipolysis and facilitating mitochondrial fatty acid uptake for β-oxidation by reducing an allosteric inhibitor, malonyl-CoA.

Accumulating evidence suggests that AMPK increases mitochondrial mass (biogenesis) by phosphorylation and activation of proliferator-activated receptor-γ coactivator 1α (PGC1α)^183^. TFEB activated by AMPK also directly binds to and activates the promoter of the gene encoding PGC1α^184^. The consequences of AMPK activation on mitochondria are not restricted to biogenesis but also include mitochondrial dynamics (fission) and mitophagy. Mitochondrial fission is mediated by dynamin-related protein 1 (DRP1), which is recruited to the outer mitochondrial membrane by mitochondrial fission factor (MFF). AMPK phosphorylates two serines on a core component of MFF and activates its action on mitochondrial fission^185^.

AMPK activates the autophagic process, including mitophagy, by two main mechanisms: i) direct activation of ULK1 and ii) inhibition of the mTOR complex. AMPK binds to and phosphorylates ULK1 on multiple residues: Ser317, Ser467, Ser555, Thr574, Ser637 and Ser777^143,186^. Activated ULK1 in turn phosphorylates class III PI3K complex I, composed of VPS34, ATG14L and Beclin1. This complex generates phosphatidylinositol-3-phosphate as a key signal for the formation of a mature phagophore, encapsulating cytosolic constituents and delivering them to the lysosome^187^. Cells expressing nonphosphorylatable ULK1 mutants accumulate defective mitochondria, supporting the importance of the AMPK-ULK1 axis for the selective removal of damaged mitochondria via mitophagy^186^.

To maintain energy and nutrient homeostasis, cells must balance anabolic and catabolic inputs. Antagonistic cross-inhibition exists between mTORC1 and AMPK signaling in energy metabolism and autophagic processes. For the inhibition of the mTORC1 complex, AMPK phosphorylates TSC2 at its Thr1127 and Ser1345 sites, which promotes the GTPase activity of the TSC1/TSC2 complexes. Rheb-GTP is transformed into an inactive Rheb-GDP state, and mTORC1 activity is turned off^188^. AMPK also directly phosphorylates the Ser772 and Ser792 sites of Raptor, increasing 14-3-3 protein binding to Raptor, hindering the binding of Raptor to mTOR or mTOR substrates, and subsequently resulting in inhibition of the mTOR signaling pathway^189^.

## Autophagic regulation on the lysosomal membrane

### Activation of mTOR and AMPK on the lysosomal membrane

As described above, the autophagosome is degraded through autophagosome-lysosome fusion and digestion by lysosomal hydrolases. This membrane fusion has been hypothesized to involve soluble N-ethylmaleimide-sensitive factor attachment protein receptor (SNARE) proteins due to their known actions in vesicle fusion^190^. The proposed molecular mechanism suggests that syntaxin 17 tethered onto the autophagosome membrane recruits synaptosomal-associated protein 29 (SNAP29) to stabilize this complex with ATG14 and vesicle-associated membrane protein 8 (VAMP8). VAMP8 is localized on endosomes and lysosomes and forms a syntaxin17-SNAP29-VAMP8 complex allowing autophagolysosomal fusion^191^. There is crosstalk between different signaling pathways, including mTORC1 and AMPK, in which the lysosome plays an important role in autophagic flux.

The localization of mTORC1 to Rab7-containing lysosomes is needed for its function, implying that the lysosome is the site of mTOR activation^115^. mTORC1 localization to the lysosome is dependent on Rag GTPases and the Ragulator complex composed of late endosomal/lysosomal adapter and MAPK and mTOR activator 1-5 (LAMTOR1-5)^116^. Rag GTPases are a Ras superfamily of small GTPases of large molecular weight lacking the post-translational modification needed for membrane localization. Instead, the Ragulator complex, which acts as a guanine nucleotide exchange factor (GEF) for RagA/B, is the anchoring site for Rag GTPases to lysosomal membrane^192^. The active conformation of Rag GTPases (RagA/B-GTP and RagC/D-GDP) with the Ragulator complex can recruit Raptor and mTORC1 to the lysosomal membrane. Activation of Rag GTPases by amino acids and glucose abundance determines lysosomal localization of mTORC1, which can be regulated by a range of GEF and GTPase-activating proteins. In addition, mTORC1 on the lysosome brings it in close proximity to its regulator, Rheb, residing on the lysosome. Thus, the lysosome provides a nutrient signaling hub that tightly controls mTORC1 activation.

**Fig. 6:**
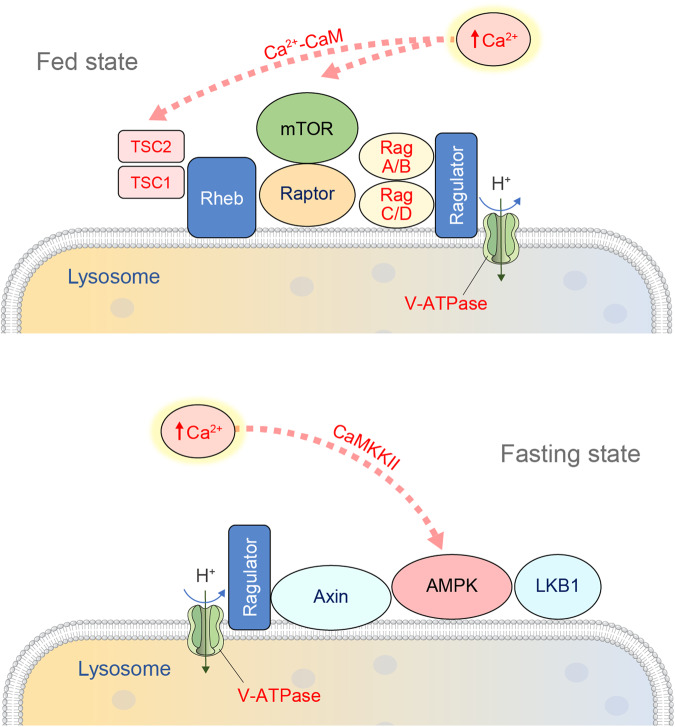
Activation of mTOR and AMPK on the lysosomal membrane. The antagonistic signals for anabolism and catabolism, mTOR and AMPK, reside on the lysosome upon activating cues. Perilysosomal Ca^2+^ elevation can simultaneously activate both mTOR and AMPK signals mediated by the Ca^2+^-calmodulin (CaM) complex and calcium/CaM-dependent protein kinase kinase 2 (CaMKKII), respectively. LKB1 liver kinase B1, Rheb Ras homolog enriched in brain, TSC Tuberous sclerosis complex.

AMPK is known to be present in the nucleus and cytoplasm but was reported to be a residential protein of the late endosome/lysosome^193^. AXIN, being a scaffold protein for AMPK and its upstream regulator LKB1, interacts with a lysosome-anchoring protein, LAMTOR1, leading to the formation of an LKB1-AMPK- AXIN-LAMTOR complex on the lysosomal membrane^116^. Starvation or the presence of AMP enhances LKB1-AMPK-AXIN binding to LAMTOR1 and accelerates AMPK activation on the lysosome (Fig. 6). Furthermore, phospho-AMPK was found exclusively on the lysosomal membrane, suggesting that LKB1 phosphorylates AMPK on this membrane surface. In addition, the energy sensor vATPase regulates lysosomal localization of the LKB1-AMPK-AXIN-LAMTOR1 complex not only for turning on catabolic processes under glucose-starved conditions but also for turning off anabolic metabolism through lysosomal association and dissociation of mTORC1^193^.

The critical role of the perilysosomal LKB1-AMPK-AXIN-LAMTOR1-vATPase axis in regulating mTORC1 and AMPK signaling was further demonstrated by its action on the metformin-induced beneficial effects on metabolism. Metformin promotes the formation of the AMPK-AXIN-LAMTOR1-vATPase complex and activates AMPK on the lysosomal membrane. In parallel, metformin treatment results in dissociation of mTORC1 from the Ragulator-vATPase complex and inhibits mTORC1 signaling^194,195^. Another cross-regulation of mTORC1 and AMPKα2 on the lysosome is mediated by sestrin2, which interacts with TSC1/2 and AMPK. Their binding inhibits Rheb-GTP loading by TSC2 phosphorylation, thus decreasing mTORC1 activity and stimulating AMPK activity^177^. Increased AMPK and suppressed mTORC1 on the lysosomal membrane can accelerate autophagic initiation, maturation, and degradation, leading to beneficial metabolic consequences.

### Perilysosomal regulation of autophagy in stressed β-cells

In most cells, Ca^2+^ signaling has been implicated in cell fate decisions, including proliferation, differentiation, migration, and cell death. In addition, Ca^2+^ regulates the autophagic process from initiation to lysosome fusion and degradation. As described above, lysosomal ion channels and transporters participate in Ca^2+^ release into perilysosomal microdomains, which can trigger important regulatory signaling in autophagy: (1) CaM-dependent AMPK and mTOR activation and (2) calcineurin-mediated TFEB/TFE3 translocation to the nucleus.

Increases in cytosolic Ca^2+^ levels, particularly near the lysosomal membrane, result in efficient phosphorylation and activation of AMPK by CaMKK2. Ca^2+^-induced activation of AMPK triggers autophagy induction by ULK1/2 phosphorylation and mTORC1 inhibition. AMPK suppresses mTORC1 via phosphorylation of TSC2 and Raptor. At the same time, elevation of perilysosomal Ca^2+^ can also activate mTORC1, which is an autophagic suppressor signal (Fig. 6). Hence, activation of mTORC1 could be prevented by Ca^2+^ chelators or TRPML1 depletion^122^. Blocking the interaction between mTOR and CaM by a CaM antagonist prevents mTORC1 activation, confirming the Ca^2+^/CaM-dependent mechanism. Thus, perilysosomal Ca^2+^ elevation activates both AMPK and mTORC1 signals mediated by CaM and CaM-dependent kinases. However, it is unclear how the activities of AMPK and mTORC1 are finely regulated through antagonistic suppression by each other under the same perilysosomal Ca^2+^ control.

As previously mentioned, lysosomal Ca^2+^ release can induce the activation of calcineurin, triggering TFEB/TFE3 dephosphorylation and nuclear translocation. TFEB and TFE3, as transcription factors and master regulators for lysosomal proteins, strongly upregulate autophagy-related gene expression and lysosomal biogenesis^196^. This signal effectively removes dysfunctional and ROS-producing mitochondria via mitophagy, since ROS trigger TRPML1-mediated Ca^2+^ release from the lysosome^88^. This mechanism is critical for β-cell function because insulin secretion relies on the synthesis of ATP and other coupling factors from mitochondrial metabolism^197,198^.

Mitochondrial stressors such as rotenone or oligomycin/antimycin increase mitophagy as a compensatory and protective process for survival. This phenomenon could be, in part, mediated by nuclear translocation of TFEB, which is stimulated by calcineurin or AMPK but inhibited by mTORC1. All these signals can be activated by Ca^2+^ increase, while BAPTA-AM, a membrane-permeable Ca^2+^ chelator, abrogates mitochondrial stressor-triggered TFEB translocation and mitophagy activation in β-cells^199^. A calcineurin inhibitor suppresses TFEB translocation and mitophagy and aggravates mitochondrial dysfunction induced by mitochondrial stressors^200^. Inhibition of TRPML1 decreases lysosomal Ca^2+^ release and mitophagy by mitochondrial stressors. Scavenging of mitochondrial superoxide also prevents mitochondrial stressor-mediated Ca^2+^ elevation and mitophagy, demonstrating the role of ROS in lysosomal Ca^2+^ release via TRPML1 in pancreatic β-cells.

## Future strategies related to perilysosomal calcium regulation against beta cell lipotoxicity

In the pathogenic progression of type 2 diabetes, the most important determining step could be the β-cell failure to compensate for the elevated insulin need. Notably, high glucose and fatty acids produce noxious oxidative stress and deleterious consequences leading to β-cell death. Accumulated evidence suggests that oxidative stress from persistent elevated fatty acids induces ER Ca^2+^ dysregulation closely connected to mitochondrial dysfunction and defective lysosomal degradative capacity. Lysosomal function is critical for autophagic flux, including mitophagy, which is an essential quality control mechanism required for cell survival against mitochondrial or metabolic stresses in β-cell lipotoxicity.

Lysosomal biogenesis and autophagic activity are stimulated by TFEB/TFE3, as demonstrated by genetic ablation of TFEB in β-cells, which causes marked aggravation of glucose intolerance and impaired insulin secretion by a high-fat diet^199^. Potentiation of perilysosomal Ca^2+^-mediated TFEB activation could be a promising therapeutic strategy to augment autophagy, counteracting organellar dysfunction due to lipotoxic stress in β-cells. It is noteworthy that the whole process of autophagy is oppositely regulated by AMPK and mTOR at the lysosomal membrane, both of which are key sensors for bioenergetic and nutritional status. Until now, the molecular mechanism governing the predominance of signaling between AMPK and mTOR has not been elucidated. Different environmental factors, including the amount and duration of cytosolic Ca^2+^ rise, crosstalk with other cell signals, or accompanying oxidative stress, can affect this predominance and autophagic outcomes. Interestingly, reducing cellular Ca^2+^ overload by Ca^2+^ channel blockers (CCB) increases autophagic activity and cell survival against lipotoxicity in HepG2 cells as well as β-cells^54,201^. Consistently, verapamil, as a frequently prescribed CCB for hypertension, promotes endogenous β-cell function and therapeutic effects in patients with recent-onset type 1 diabetes^202^. Recently, we demonstrated that the small molecule ER Ca^2+^ pump activator CDN1163 increases ER Ca^2+^ content, improves mitochondrial function, and protects against palmitate-induced lipotoxicity in pancreatic β-cells^203^. We propose that therapeutic strategies to activate ER Ca^2+^ pumps could correct perilysosomal Ca^2+^ overload and mitigate autophagic defects induced by sustained Ca^2+^ elevations. In addition, recovering mitochondrial function by promoting organellar Ca^2+^ uptake could be another effective strategy to improve autophagy and protect β-cells from lipotoxicity.
