# Author Correction: Regulation of autophagy by perilysosomal calcium: a new player in β-cell lipotoxicity

**DOI:** 10.1038/s12276-024-01265-4

**Published:** 2024-06-11

**Authors:** Ha Thu Nguyen, Andreas Wiederkehr, Claes B. Wollheim, Kyu-Sang Park

**Affiliations:** 1 Department of Physiology, Yonsei University Wonju College of Medicine, Wonju, Korea; 2 Mitohormesis Research Center, Yonsei University Wonju College of Medicine, Wonju, Korea; 3 Ecole Polytechnique Fédérale de Lausanne, Lausanne, Switzerland; 4 Department of Cell Physiology and Metabolism, University of Geneva, Geneva, Switzerland; 5 Department of Clinical Sciences, Lund University, Malmö, Sweden

**Keywords:** Type 2 diabetes, Energy metabolism

Correction to: *Experimental & Molecular Medicine* 10.1038/s12276-024-01161-x, published online 01 February 2024

After online publication of this article, the authors noticed an error in the Acknowledgements section.

The correct statement of this article should have read as below.

“This work was supported by the Myung Sun Kim Memorial Foundation (2016), Yonsei University, Bio & Medical Technology Development Program (2020M3A9D8039920) from the Ministry of Science and ICT, Korea. We would like to thank Luong Dai Ly, Da Dat Ly, and Pedro Gabriel Coffler Zorzal for their valuable support of this manuscript.”

The authors apologize for any inconvenience caused.

The original article has been corrected.

